# The Global Secondary Metabolite Regulator *AcLaeA* Modulates *Aspergillus carbonarius* Virulence, Ochratoxin Biosynthesis, and the Mode of Action of Biopesticides and Essential Oils

**DOI:** 10.3390/toxins17010002

**Published:** 2024-12-24

**Authors:** Maria K. Iliadi, Maria Varveri, Anastasia E. Kapetanakou, Panagiotis N. Skandamis, Dimitrios I. Tsitsigiannis

**Affiliations:** 1 Laboratory of Plant Pathology, Department of Crop Science, Agricultural University of Athens, 118 55 Athens, Greece; maria_iliadi90@hotmail.com (M.K.I.); varvmar@gmail.com (M.V.); 2 Institute of Technology of Agricultural Products, Hellenic Agricultural Organization—DIMITRA (ELGO-DIMITRA), 141 23 Lykovrissi, Greece; akapetanakou@elgo.gr; 3 Laboratory of Food Quality Control and Hygiene, Department of Food Science & Technology, Agricultural University of Athens, 118 55 Athens, Greece; pskan@aua.gr

**Keywords:** *Aspergillus carbonarius*, secondary metabolism, ochratoxin A, mycotoxins, essential oils, biopesticides, biological plant protection products, AcleaA

## Abstract

*Aspergillus carbonarius* is considered one of the main fungi responsible for black and sour rot in grapes, as well as the production of the carcinogenic mycotoxin ochratoxin A. The global regulatory methyltransferase protein *LaeA* controls the production of various secondary metabolites in *Aspergillus* species, as well as influences sexual and asexual reproduction and morphology. The goal of this study was to investigate the role of the regulatory gene *AclaeA* in physiology, virulence, and ochratoxin A (OTA) production by deleting this gene from the genome of a wild-type *A. carbonarius* strain. The evaluation data on the morphological characteristics, virulence experiments in three different grape varieties, and OTA analysis of Δ*AclaeA* mutants showed that the growth and the OTA production by Δ*AclaeA* strains were significantly reduced. The mutant strains were also less virulent, producing 40–50% less conidia in three different cultivars of grape berries. Additionally, the gene *AclaeA* was considerably repressed after the application of three commercial biopesticides (Trianum-P^®^, Vacciplant^®^, and Serenade^®^ Max) and the essential oils (EOs) cinnamon, geranium, and thyme, which were also shown to inhibit OTA biosynthesis in *A. carbonarius*. The study of the regulatory gene *AclaeA* can contribute to a broader understanding of the role of secondary metabolites during *A. carbonarius*—grape interactions, as well as the discovery of the mode of action of biological plant protection products and EOs against this mycotoxigenic fungus.

## 1. Introduction

Black Aspergilli are responsible for causing black and sour rot on grape berries and are also the main source of ochratoxin A (OTA) contamination [1]. Among black Aspergilli, *Aspergillus carbonarius* is one of the most notorious OTA-producing species [2]. OTA poses a threat to both human and animal health, as it was reported that it causes Balkan Endemic Nephropathy (BEN) and has teratogenicity, immunosuppressive, genotoxicity, cytotoxicity, and carcinogenicity effects [3]. Hence, the International Agency for Research on Cancer (IARC) has designated OTA as a group 2B carcinogen. In addition, during the synthesis of OTA, *A. carbonarius* goes through morphological and metabolic changes that cause oxidative stress to the fungus, resulting in the production of sesquiterpenes. These volatile molecules may affect grape products’ quality and acceptability by consumers, depending on their concentration [4,5]. At present, the European Union has established strict regulations, setting maximum levels for OTA of 2 ppb in wine, must, and grape juice and 10 ppb for dried vine fruits (Commission regulation No. 1881/2006).

The virulence mechanism of *A. carbonarius* and the involvement of OTA in its infection strategy remain poorly understood. Nonetheless, engineering the global regulatory network that governs the expression and metabolite production in filamentous fungi has been the main objective of numerous studies. Bok and Keller (2004) identified the *laeA* gene as a global regulator of secondary metabolism in *A. nidulans*, *A. terreus*, and *A. fumigatus*, among other fungi. The *laeA* gene was named as an abbreviation of ‘loss of aflatoxin-related (*AflR*) gene expression’ after the mechanism in aflatoxin biosynthesis that was discovered to be regulated by the *laeA* gene. In *A. nidulans*, *laeA* encodes a nuclear methyltransferase protein that is required for the expression of secondary metabolite genes, while its presence is considered indispensable for mycotoxin, antibiotic, and mycelial pigment biosynthesis [6]. *LaeA* has a global regulatory effect not only on secondary metabolites but also on asexual and sexual reproduction and morphology [7]. In Aspergilli, *LaeA* is proposed to carry out regulation at the level of histones by regulating clusters of genes encoding secondary metabolites, which are often localized near the telomeres of chromosomes. Because of the co-clustering of genes for secondary metabolites, *LaeA* has the potential to regulate a multitude of these genes. Linde et al. (2016) proved that the knockout of *laeA* in *A. carbonarius* reduced the synthesis of endoglucanases and citric acid substantially, but not that of beta-glucosidases or xylanases, indicating the role of *AclaeA* in citric acid and endoglucanases production by this fungal species. In Δ*laeA* null mutants, there was also a considerable reduction in OTA production, ranging from 68.5% to 99.4% [8]. This was linked to the downregulation of a non-ribosomal peptide synthetase that is involved in the biosynthesis of OTA in a study by Crespo-Sempere et al. (2013) [9]. However, the role of the *laeA* gene in *A. carbonarius* virulence and pathogenicity in plants has not been extensively investigated till now.

Yeasts and bacteria, particularly lactic acid bacteria (LAB) due to their capacity to proliferate and persist in food products under a variety of environmental conditions, either as a component of the native microflora or supplemented as a starter or protective cultures, represent promising potential biocontrol agents of fungal growth and/or OTA occurrence [10,11]. The results obtained from a study by Zhu et al. (2015) showcased that the inhibitory effect on fungal growth and OTA production by beneficial microorganisms against *A. carbonarius* and *A. ochraceus* was likely explained by the production of an antifungal metabolite, in the case of the yeasts, or by the competition for nutrients and space by the beneficial fungi and yeasts [12]. Polyketide synthase, non-ribosomal peptide synthase, monooxygenase, and the regulatory genes *laeA* and *veA* were all found to be downregulated in both *A. carbonarius* and *A. ochraceus* after exposure to yeast volatile organic compounds (VOCs) [13]. Nonetheless, an inhibitory effect on *AclaeA* expression by beneficial microbes included in registered biopesticides has not yet been reported.

Plant extracts also seem to be a promising tool for controlling fungal contamination in food commodities [14]. Cistus and orange peel extracts have demonstrated high antifungal activity and significantly reduced mold symptoms by *A. carbonarius* on grapes according to Chtioui et al. (2023) [15]. The results obtained from the same study showed that while there was no impact on fungal growth, eucalyptus extract exhibited a significant capacity in suppressing OTA production by the fungus, by up to 80%. In addition, it has been established that the essential oils of clove, anise, boldo, poleo, and thyme possess strong antifungal properties and can effectively regulate the production of OTA in *Aspergillus* spp. [16]. Thus far, there have been few assessments demonstrating the impact of essential oils (EOs) on the expression of *A. carbonarius* genes that have been correlated with OTA biosynthesis. For example, essential oils of cardamom, fennel, chamomile, rosemary, anise, and celery reduced the expression of the genes *acpks*, *acOTApks*, *acOTAnrps*, *laeA*, and *veA*, which have been linked to the synthesis of the enzymes needed for OTA biosynthesis in *A. carbonarius* [17]. Finally, eugenol was found to significantly downregulate five genes in the OTA biosynthesis gene cluster, including the global regulator *laeA* [18].

Given the upcoming challenges of climate change and the increase in mycotoxin contamination, the main objectives of this study were to investigate the role of the *laeA* gene as a regulator of secondary metabolism and as a pathogenicity and/or virulence factor in *A. carbonarius*. Additionally, the goals of this study were to identify effective biological Plant Protection Products (bioPPPs) and Essential Oils (EOs) that target the downregulation of *AcLaeA* expression and therefore the inhibition of OTA biosynthesis. Knowledge of the mode of action of the aforementioned EOs and bioPPPs in OTA reduction is required since such information could possibly give insight into the successful prevention of fungal growth and OTA contamination.

## 2. Results

### 2.1. AclaeA Gene Analysis

BLAST analysis of the genome of *A. carbonarius* with the *laeA* gene of *A. nidulans* resulted in the presence of an orthologous gene named *AclaeA*. The *AclaeA* sequence was 1213 bp, including the start and stop codon and one intron. The sequence was predicted to encode a sequence of 341 amino acids. For validation, the amino acid sequence was used as a query in a protein–protein blast that returned numerous hits with high similarity to the query; the majority were LaeA methyltransferases. The result with the highest similarity to the LaeA amino acid sequence from *A. carbonarius* was the methyltransferase LaeA from *Aspergillus parasiticus*, with an identity of 78%, E value of 0.0, and a query cover of 99%.

### 2.2. Validation of AcLaeA Deletion Strains

The *AclaeA* gene was deleted in the ochratoxigenic *A. carbonarius* Ac-5010 strain by targeted gene replacement using *Agrobacterium tumefaciens*-mediated transformation. Using specific primers, two genome sequences of about 1000 bp before the start and stop codon, respectively, of *AclaeA* were amplified and subcloned into the pBluescript plasmid vector. Between these two regions, the geneticin cassette was subcloned in order to replace the *AclaeA* gene after transformation. The deletion *AclaeA* construct was transferred to the binary vector pGKO2 and then incorporated using the Ti plasmid of *Agrobacterium tumefaciens* via a double recombination event into the wild-type *A. carbonarius* strain 5010, creating the mutant Δ*AclaeA*. A total of 20 putative Δ*AclaeA* mutant transformants were recovered, and 10 mutants were confirmed by PCR to have a disrupted *AclaeA* gene. The entire ORF of *AclaeA* gene was successfully deleted in Δ*AclaeA* mutants, as proven by PCR analysis (Figure S1). For the knockout transformants, PCR primers were designed to check if the geneticin was correctly inserted inside the *AclaeA* gene, disrupting the gene’s transcription, resulting in a PCR product of 1.45 kb in size (Figure S1).

### 2.3. AclaeA Gene Expression

**Figure 1:**
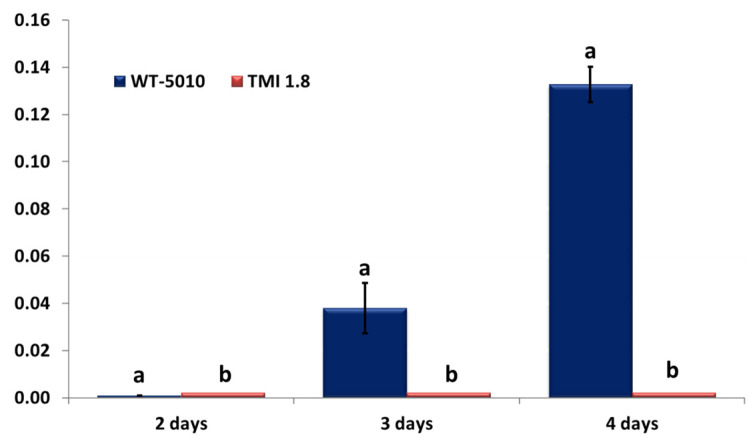
Expression of *AclaeA* gene normalized with beta-tubulin gene expression in the Δ*AclaeA* TMI 1.8 and wild-type Ac-5010 strains. The statistical analysis was performed for each day separately, and the different letters indicate the statistically significant differences between the wild strain Ac-5010 and the Δ*AclaeA* strain TMI1.8 after one-way ANOVA followed by Tukey’s multiple comparison post hoc test (*p* < 0.05).

The study of the expression of the *AclaeA* gene was performed on the wild-type strain Ac-5010 and Δ*AclaeA* strain TMI1.8. The results showed that the expression of the *AclaeA* gene in the Ac-5010 wild-type strain initiated on the 2nd day of the fungal culture and increased significantly on the 4th day, while, as expected, the mutant TMI1.8 showed no expression of the *AcLaeA* gene that confirms the successful deletion of this gene from its genome (Figure 1).

### 2.4. Morphology and Physiology of ΔAclaeA Phenotypes

**Figure 2:**
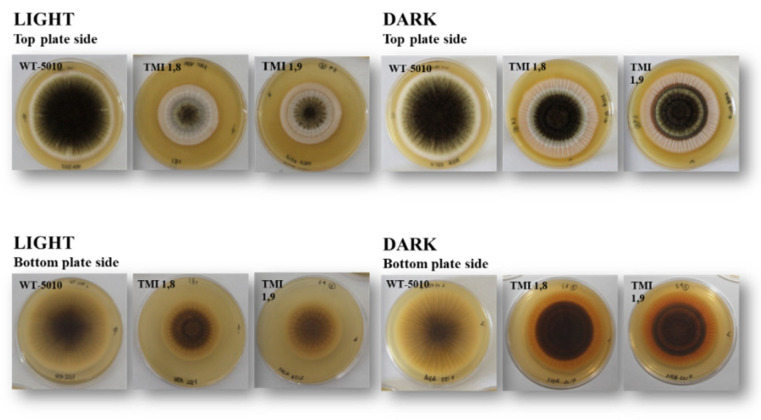
Phenotypic analysis of Δ*AclaeA* strains. Phenotypes of Δ*AclaeA* strains in MEA media point inoculated on plates, incubated for 8 days at 28 °C under light and dark conditions. Front (**top**) and reverse (**bottom**) colony view of the wild-type Ac-5010 strain and Δ*AclaeA* strains.

In order to assess the physiological characteristics (growth and colony morphology) of Δ*AclaeA* mutants compared to the Ac-5010 wild type, all strains were grown in non-selective media (MEA plates) under light and dark conditions. Three Δ*AclaeA* mutants, TMI1.4, TMI1.8, and TMI1.9, were used. The colonies of Δ*AclaeA* mutants consisted of a compact white basal mycelial colony covered by a layer of dark black conidia heads showing marked radial grooves that were not present in the wild-type strain. Another interesting finding was the concomitant presence of exudates from the colonies when the Δ*AclaeA* mutants TMI1.4, TMI1.8, and TMI1.9 were incubated in darkness (Figure 2).

**Figure 3:**
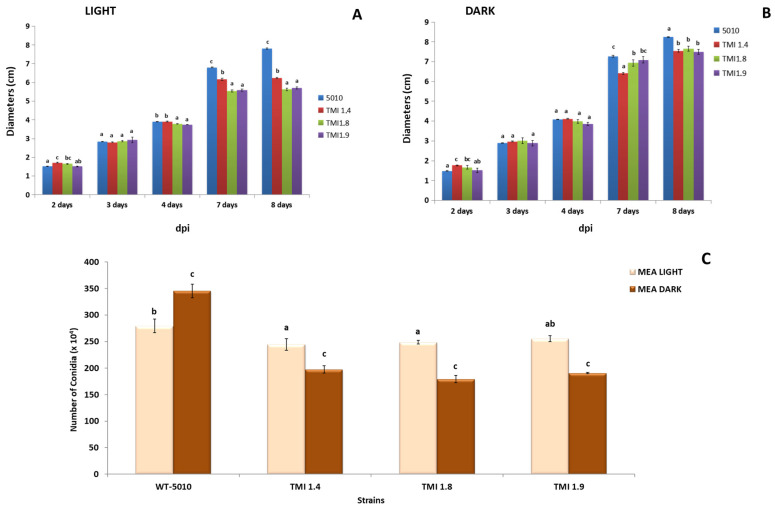
Growth of strains in media. Mycelial growth of Δ*AclaeA* and wild-type Ac 5010 strains inoculated in MEA plates incubated for 8 days at 28 °C under dark (**A**) and light (**B**) conditions (**C**). Conidia production per mm^2^ of colony in the Δ*AclaeA* and wild-type 5010 strains. The statistical analysis was performed for each day separately, and the different letters indicate the statistically significant differences between the wild strain Ac 5010 and the Δ*AclaeA* strains after one-way ANOVA followed by Tukey’s multiple comparison post hoc test (*p* < 0.05).

The growth of the strains was also assessed by measuring the colony diameter of the strains during eight days of cultivation. The colony diameter of all Δ*AclaeA* strains was smaller compared to the Ac-5010 wild type under both light and dark conditions. After eight days of incubation, the colony diameter of the mutants was between 1.6 and 2.2 cm smaller than the wild-type diameter under light and 0.6 and 0.8 cm under darkness conditions (Figure 3). Deletion of the *AclaeA* gene also resulted in a reduction in conidial production under light and dark conditions (Figure 3C). When the Δ*AclaeA* strains were cultivated under light conditions, the reduction was 13% compared to the wild-type strain. In dark conditions, the Δ*AclaeA* strains produced 48% less conidia than the wild-type strain. We also observed a positive effect of the dark conditions on the conidiation in the wild-type strain. It was found that the wild-type strain produced 20% more conidia under dark conditions.

### 2.5. Pathogenicity and Virulence Tests of ΔAclaeA Strains on Grapes

**Figure 4:**
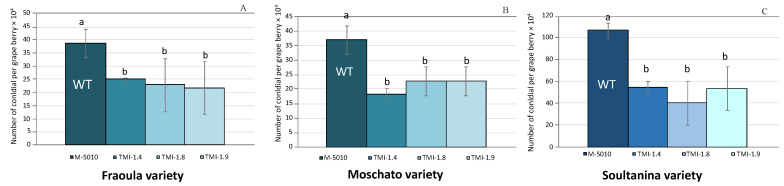
Conidia production of the Δ*AclaeA* and wild-type Ac 5010 strains in grape varieties *cv*. Fraoula (**A**), *cv*. Moschato (**B**), and *cv*. Soultanina (**C**) after inoculation for 4 days at 28 °C under 12 h photoperiod. Letters indicate homogeneous groups (ANOVA, *p* < 0.05). Statistical analysis was performed using one-way ANOVA followed by Tukey’s multiple comparison post hoc test (*p* < 0.05). Letters above the graphs indicate differences between treatments (*p* < 0.01).

**Figure 5:**
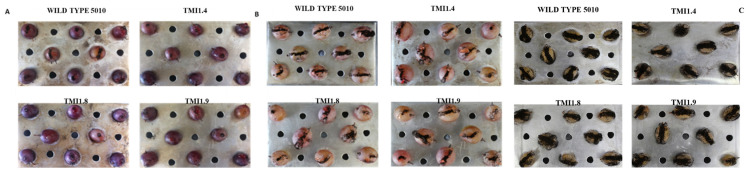
Phenotypes of Δ*AclaeA* and wild-type Ac 5010 strains in the varieties Moschato (**A**), Fraoula (**Β**), and Soultanina (**C**), three days post-inoculation and incubation in 28 °C under 12 h photoperiod.

To further elucidate the results of the deletion of the *AclaeA* gene from the *A. carbonarius* genome, pathogenicity tests were carried out. Four days after grapes’ infection on three different red and white grape varieties, the conidia were measured using a hemocytometer. Δ*AclaeA* strains produced about 40–50% less conidia compared to the control grapes in all three varieties. We also observed that the *cv*. Soultanina grape variety was more sensitive than *cv*. Fraoula and *cv*. Moschato varieties. These results indicate that the *AclaeA* gene plays a significant role in the virulence mechanisms of *A. carbonarius* (Figure 4 and Figure 5).

### 2.6. In Vitro Ochratoxin Production of ΔAclaeA Strains

**Figure 6:**
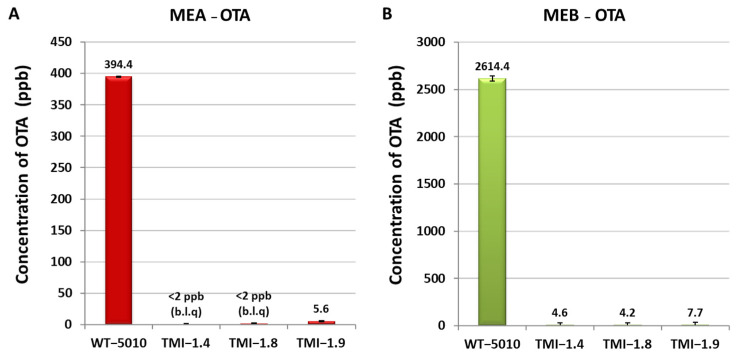
OTA production in Δ*AclaeA* and wild-type 5010 strains inoculated in MEA plates (**A**) and MEB (**B**), incubated for 7 days at 25 °C under dark conditions (b.l.q. = below limit of quantification).

The effect of deletion of the *AclaeA* gene was also evaluated in OTA biosynthesis in *A. carbonarius* strains. The production of OTA was quantified via HPLC and was decreased drastically in Δ*AclaeA* strains compared to wild-type strain Ac-5010 on both (MEA and MEB) substrates. On the MEA medium, the wild strain produced 394.4 ppb, whereas on Δ*AclaeA* strains TMI 1.4 and TMI 1.8, the OTA production was below the limit of quantification (2 ppb) of the method. In strain TMI 1.9, the production of OTA was 5.6 ppb. In the MEB medium, the wild strain Ac-5010 produced 2614.4 ppb, whereas the Δ*AclaeA* strains TMI1.4, TMI1.8, and TMI1.9 produced 4.6, 4.2, and 7.7 ppb, respectively (Figure 6). These results indicate that the *AclaeA* gene is essential for OTA production in *A. carbonarius*.

### 2.7. Expression of AclaeA Post Biocontrol Product Application

**Figure 7:**
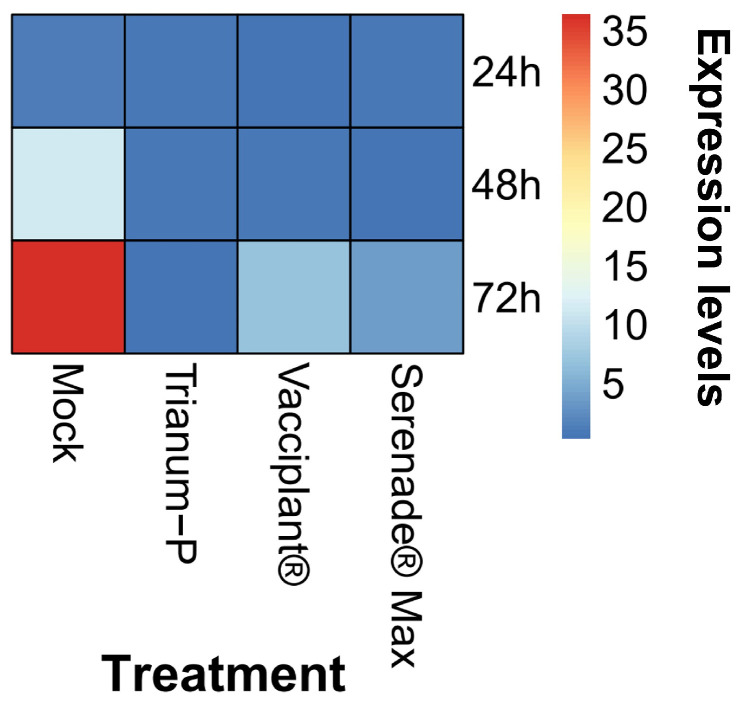
*AcLaeA* is repressed after the application of the biopesticides Trianum-P^®^, Vacciplant^®^, and Serenade^®^, as revealed by qRT-PCR. Heatmap shows the quantification of *AclaeA* expression level at each time point and was determined by first normalizing the target RNA to the internal standard RNA (tubulin).

The expression of *AclaeA* was examined after the treatment of fungal cultures with three biopesticides: Vacciplant^®^ (laminarin), Trianum-P^®^ (*Trichoderma harzianum* strain T22), and Serenade^®^ Max (*Bacillus amyloliquefaciens* QST 713). The selected biocontrol products were able to successfully reduce the sour rot disease and OTA contamination in vines in several field experiments carried out by the authors (unpublished data), and the goal of this study was to examine the potential of these formulations to regulate the expression of the *AclaeA* gene in *A. carbonarius*. Experiments were performed in nutrient liquid media in vitro, and the *AclaeA* gene expression was examined after 48 h, 72 h, and 96 h, conditions commonly found to induce the expression of the gene (Figure 1). Real-time qRT-PCR analysis of the transcriptional kinetics of *AclaeA* that occurs after treatment of the biopesticides compared with mock inoculation is shown in Figure 7. The expression of *AclaeA* was steadily increased for the control treatments from 0 h to 72 h. At 24 h, transcription of *AclaeA* increased, reaching an approximately 10-fold increase, and, at 48 h, an approximately 32-fold increase. In sharp contrast, Trianum-P repressed *AclaeA* expression by 16- and 24-fold at 48 h and 72 h post-inoculation, respectively. Vacciplant^®^ downregulated *AclaeA* expression by 12- and 5-fold, whereas Serenade^®^ Max repressed the gene expression by 38- and 9-fold at 48 h and 72 h, respectively (Figure 7). These data indicate clearly that the three biopesticides repress *AclaeA* expression, suggesting a possible mechanism of their active ingredients in OTA reduction in field vineyard studies.

### 2.8. In Vitro Sensitivity Tests of Ten Essential Oils Against Aspergillus carbonarius

The role of ten EOs was investigated in the growth and OTA biosynthesis in *A. carbonarius.* The results demonstrated high variability regarding the inhibitory effect of EOs in *A. carbonarius* mycelial growth (Table 1). Geranium, thyme, and cinnamon EOs in MEA led to EC_50_ values that ranged from 1 μL mL^−1^ to 8.3 μL mL^−1^, which suggests that these extracts are effective in inhibiting the mycelial growth of the WT strain Ac-5010, followed by citronella and mint, with EC_50_ values ranging between 13.07 μL mL^−1^ and 16.90 μL mL^−1^. The less effective EOs were lavender and sage, with EC_50_ values of 48.82 μL mL^−1^ and 61.67 μL mL^−1^, respectively. Accordingly, in CYA medium, thyme and cinnamon demonstrated EC_50_ values that did not surpass 3.35 μL mL^−1^, while cinnamon extract completely inhibited the growth of the fungus, at all concentrations tested, leading to EC_50_ value below the minimum dose applied (<2 μL mL^−1^). EC_50_ value for citronella oil was 9.02 µL mL^−1^, while rosemary EO showed no efficacy in inhibiting Ac-5010 mycelial growth since the EC_50_ value surpassed the maximum applied dose, which was 100 μL mL^−1^ in CYA medium (Table 1).

Regarding the Greek endemic *A. carbonarius* strain Ac-29, geranium, thyme, and cinnamon EOs demonstrated the lowest EC_50_ values in MEA, between 2.77 μL mL^−1^ and 11.75 μL mL^−1^, similar results as those obtained from Ac-5010 in the same substrate. Additionally, EC_50_ values for citronella and mint were approximately 10.98 μL mL^−1^ and 13.86 μL mL^−1^ for Ac-29, results that were in accordance with the EC_50_ values obtained from Ac-5010. In sharp contrast, tea tree, lavender, marjoram, sage, and rosemary EOs resulted in high EC_50_ values that ranged from 50.04 μL mL^−1^ to >100 μL mL^−1^.

Regarding the inhibition of conidia production by the Ac-5010 wild-type strain, application of the highest concentration of the tested EOs rosemary, thyme, mint, geranium, marjoram, tea tree, and citronella resulted in a 100% reduction in conidia produced by the fungus, while cinnamon demonstrated 100% inhibition of conidia production in multiple doses tested, which ranged between 3 μL mL^−1^ and 100 μL mL^−1^. As for the CYA substrate, mint, geranium, tea tree, thyme, and citronella led to 100% inhibition of conidia produced by the wild-type strain, Ac-5010, in the maximum applied dose tested, while cinnamon generated total inhibition of conidia production in multiple concentrations that also ranged between 3 μL mL^−1^ and 100 μL mL^−1^.

The results obtained for the Ac-29 strain were in accordance with the results obtained for the wild-type strain Ac-5010. More specifically, thyme, mint, geranium, and citronella inhibited conidia production in MEA at rates that reached 100% with the maximum dose applied, while cinnamon effectuated 100% inhibition of conidia production in all tested concentrations between 3 μL mL^−1^ and 100 μL mL^−1^. Regarding the CYA substrate, mint, geranium, tea tree, thyme, and citronella reduced conidia produced by the strain Ac-29 at rates that reached 100%, while cinnamon resulted in 100% inhibition of conidia production when applied in doses of 3 μL mL^−1^, 6 μL mL^−1^, 20 μL mL^−1^, and 100 μL mL^−1^.

### 2.9. Effectiveness of EOs in Reducing OTA Production

**Figure 8:**
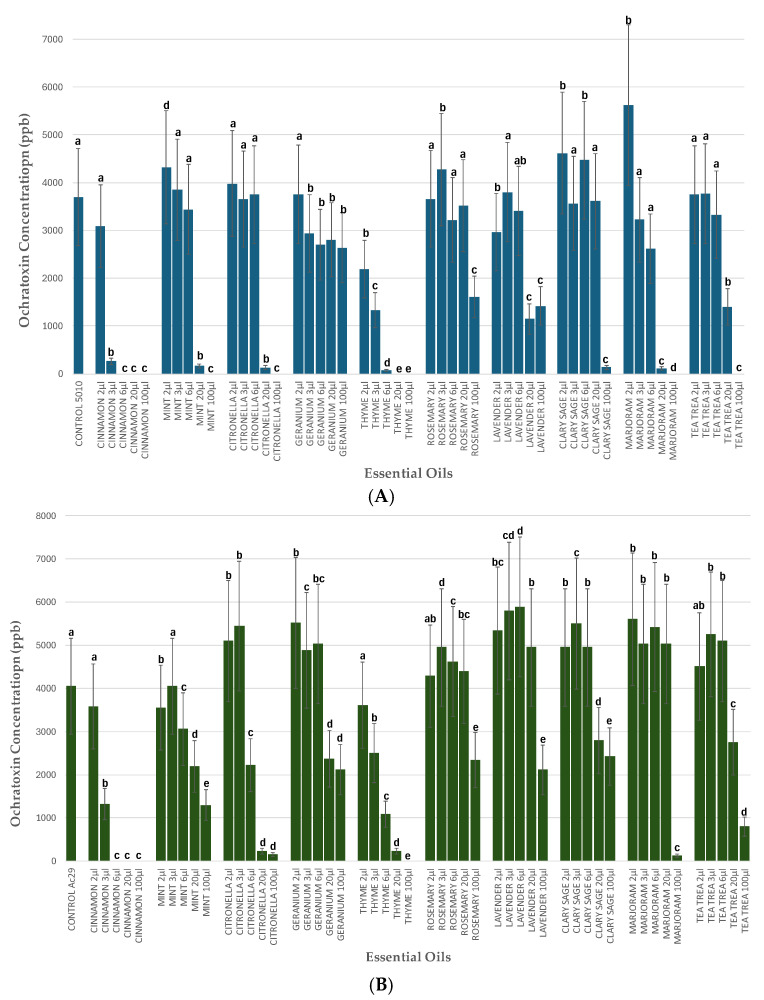
OTA produced (ppb) by *A. carbonarius* strains Ac-5010 (**A**) and Ac-29 (**B**) in MEA plates amended with different concentrations of each EO concentration. The statistical analysis was performed for each EO separately compared to the non-amended plate. The different letters indicate statistically significant differences between the different concentrations of each EO and the control after one-way ANOVA followed by Tukey’s multiple comparison post hoc test (*p* < 0.05).

The evaluation of EOs for their ability to reduce OTA production by the *A. carbonarius* strains Ac-5010 and Ac-29 was carried out on the 7th day post-inoculation in MEA plates using the enzyme-linked immunosorbent assay (ELISA) based on the AgraQuant^®^ ELISA Ochratoxin Assay kit protocol (2–40 ppb) (Figure 8).

Considering the produced OTA by the wild-type strain Ac-5010, cinnamon, mint, thyme, tea tree, marjoram, and citronella at the maximum dose tested resulted in a 100% reduction in OTA biosynthesis, while the same result was observed when 6 μL mL^−1^, 20 μL mL^−1^, and 100 μL mL^−1^ of cinnamon extract and 20 μL mL^−1^ of thyme were applied. Lower concentrations of mint (20 μL mL^−1^), cinnamon (3 μL mL^−1^), citronella (20 μL mL^−1^), thyme (6 μL mL^−1^), clary sage (100 μL mL^−1^), and marjoram (20 μL mL^−1^) also led to a significant reduction in OTA from 93% to 98%. In other concentrations, thyme (2 μL mL^−1^, 3 μL mL^−1^), rosemary (100 μL mL^−1^), lavender (20 μL mL^−1^ and 100 μL mL^−1^), and tea tree (20 μL mL^−1^) reduced OTA by 41% to 69%, whereas geranium (6 μL mL^−1^ and 100 μL mL^−1^) led to a <50% OTA reduction (Figure 8A).

Accordingly, regarding the strain Ac-29 (Figure 8B), cinnamon and thyme at the maximum tested concentration (100 μL mL^−1^), effectuated a 100% reduction in OTA compared to the control. Cinnamon also led to a 100% reduction in OTA when 6 μL mL^−1^ and 20 μL mL^−1^ of its EO was applied to MEA plates, which sets cinnamon as the most effective EO for both strains at the lowest concentration of 6 μL mL^−1^. Citronella (20 μL mL^−1^ and 100 μL mL^−1^), thyme (20 μL mL^−1^), and marjoram (100 μL mL^−1^) also led to a significant reduction in OTA from 94 to 97%. Other concentrations of cinnamon (3 μL mL^−1^), mint (100 μL mL^−1^), thyme (6 μL mL^−1^), and tea tree (100 μL mL^−1^) reduced OTA by 67% to 80%. The rest of the EOs concentrations led to a <50% OTA reduction (Figure 8B).

### 2.10. Effectiveness of EOs in Suppressing AclaeA Expression

**Figure 9:**
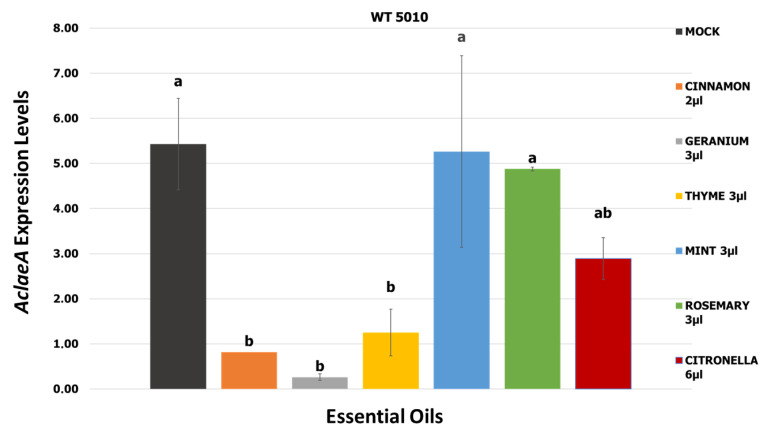
*AcleaA* expression levels post-EO application at specific concentrations in MEA medium, including expression levels for the control of the experiment (mock with wild-type strain Ac-5010). Statistical analysis was performed using one-way ANOVA followed by Tukey’s multiple comparison post hoc test (*p* < 0.05). Letters above the graphs indicate differences between treatments (*p* < 0.01).

Selected EOs and the corresponding lower concentrations that demonstrated a significant inhibition rate considering mycelial growth, conidia, and OTA production by *A. carbonarius* strains were subjected to an in vitro evaluation of their ability to repress *AclaeA* gene expression. Real-time qRT-PCR analysis of the transcriptional kinetics of *AclaeA* was carried out four days post-inoculation of MEA plates with the wild-type strain Ac-5010. As shown in Figure 9, geranium, cinnamon, and thyme, when applied at 3 μL mL^−1^, 2 μL mL^−1^, and 3 μL mL^−1^ concentrations, respectively, resulted in significantly lower *AclaeA* expression compared to the control of the experiment (MEA plates inoculated with the wild-type strain Ac5010), suggesting a possible mode of action of the aforementioned EOs in reducing OTA production by the fungus. On the contrary, mint and rosemary applied at the concentration of 3 μL mL^−1^ did not have any significant effect on *AclaeA* gene expression levels in comparison to the control of the experiment (Figure 9).

## 3. Discussion

In order to find new, alternative methods to effectively control the black and sour rot on grapes and prevent OTA contamination in wine and grape products, we tried to identify factors that affect the pathogenicity and/or virulence of the fungus *A. carbonarius*. The present study was based on the regulation of the secondary metabolism of the fungus *A. carbonarius* and to find a possible interaction between secondary metabolism and pathogenicity/virulence of the fungus. *AcLaeA,* a transcriptional factor regulating the production of secondary metabolites, is a polyketide synthase in several *Aspergillus* species, and it is required not only for the biosynthesis of mycotoxins (e.g., sterigmatocystin, aflatoxin, penicillin, and gliotoxin) but also mycelial pigments in *A. nidulans* and *A. fumigatus* [19]. A global regulatory role for *LaeA* in Aspergilli is suggested by the observation that the same gene regulates the synthesis of three distinct categories of chemicals, including organic acid, enzyme, and mycotoxin compounds [6,8,20].

The *AclaeA* gene that was identified and investigated in this study is an ortholog to the *A. nidulans laeA* gene. *AcLaeA* is a putative methyltransferase, and the gene was deleted successfully in the *A. carbonarius* Ac-5010 strain (Figures S1 and S2). Regarding the morphology and physiology of Δ*AclaeA* phenotypes, a decrease in conidial production on PDA and MEA plates under both light and dark conditions was observed upon its deletion. Notably, conidia reduction was higher when plates were incubated in dark (48%) compared to light conditions (Figure 2 and Figure 3). Respectively, the regulatory role that the protein methyltransferase *LaeA* poses in the sexual and asexual development of other fungal species, such as species belonging to Ascomycota like *A. niger*, *A. fumigatus,* and *Trichoderma atroviridae*, has also been confirmed by several studies [21,22,23].

The pathogenicity/virulence assays in grape berries demonstrated that the Δ*AclaeA* mutant strains are still pathogenic but with reduced severity and conidia production. Specifically, a 40–50% decrease in conidia production in different grape varieties was noticed compared to the control of the experiment. Additionally, a relative sensitivity of *cv*. Soultanina (a table grape cultivar with thin berry skin) was observed compared to the other two Greek varieties tested (*cv*. Fraoula and *cv*. Moschato).

Regarding the OTA production by the Δ*AclaeA* mutants, the HPLC results revealed a very significant decrease in OTA levels in MEA and MEB plates, where the wild-type strain produced 394.44 mg/kg and 2614.4 mg/kg of OTA in each media, respectively, while the Δ*AclaeA* mutants resulted in OTA levels between <2.0 mg/kg (limit of quantification) and 7.7 mg/kg. It has been found that in *Aspergillus* species, the expression of several metabolic gene clusters, such as those encoding the carcinogen sterigmatocystin, the antibiotic penicillin, and the antihypercholesterolemic drug lovastatin, is inhibited by the deletion of *laeA* [19]. In accordance with the current study, upon performing a functional investigation on colonized fruit, it was observed that the Δ*AclaeA* mutant strain significantly down-regulated all five genes within the OTA cluster, leading to a notable decrease in OTA synthesis [9]. The production of OTA in colonized nectarines and grapes was also significantly decreased upon deletion of *laeA* in *A. carbonarius* in a study by Maor et al. (2021) [24]. The authors explained that due to reduced regulating gluconic acid (GLA) synthesis, the Δ*laeA* mutant was unable to effectively acidify the colonized tissue, which resulted in decreased pathogenicity in infected fruit when compared to the wild type (WT) [24].

The next goal was to investigate if *AclaeA* is regulated by biopesticides that can control *A. carbonarius* and reduce OTA biosynthesis. The most effective biological control agents (BCAs) for OTA-producing fungi are thought to be antagonistic yeasts, which have been shown to be effective in controlling and/or degrading these OTA-producing fungi in several laboratory studies [25]. Regarding beneficial fungi belonging to *Trichoderma* species, their ability to reduce mycotoxins, including OTA, has been correlated with the production of peroxidase enzymes [26]. Additionally, carboxypeptidase A (CPA) was found to be responsible for OTA degradation in the presence of the endophytic fungus *Trichoderma koningii* [27]. Comparable results were presented in another study, where the removal of OTA was rendered to the carboxypeptidase and peptides present in liquid cultures of *Bacillus subtilis* CW14 [28]. *B. subtilis* is thought to be a significant biocontrol agent against a plethora of pathogenic fungi that produce toxins [29,30,31]. Nonetheless, the mode of action toward OTA reduction in the biological plant protection products used in this study, containing such microorganisms as *Trichoderma harzianum* and *Bacillus subtilis*, has not been clarified. In the current study, we discovered for the first time to our knowledge that the three tested biopesticides, Vacciplant^®^ (laminarin), Trianum-P^®^ (*T. harzianum* strain T22), and Serenade Max^®^ (*Bacillus subtilis* QST 713), significantly repress *AclaeA* expression, suggesting a possible novel mechanism of their active ingredients in OTA reduction.

Several studies indicate that plant extracts are also a promising biocontrol tool for controlling plant pathogens since they are abundant in nature, easily biodegradable, and consist of an environmental-friendly solution for controlling plant diseases [32]. EOs derived from various plants have garnered significant attention for their antimicrobial properties, particularly in the context of food safety and preservation. They also offer a plentiful supply of secondary metabolites, including phenylpropanoids and terpenoids with antimicrobial, antifungal, and antioxidant properties [33]. In these studies, in vitro tests evaluating ten EOs revealed that cinnamon thyme and geranium were the most effective in reducing the mycelial growth of *A. carbonarius*. In addition, the same three EOs reduced conidia production up to 100% in multiple doses tested. In accordance with our results, lower amounts of cinnamon could completely prevent fungal growth in each of the solid media examined in a study by Lappa et al. (2017) [34], indicating cinnamon to be the most effective EO in inhibiting *A. carbonarius* growth. In a similar study, dittany, oregano, thyme, and savory, which have carvacrol and/or thymol as a common component, significantly reduced or even inhibited the growth of the fungus in all treatments when high concentrations were applied [35]. As can be seen in this study, the efficacy of EOs can be influenced by various factors, including the concentration, substrate, method of application, and the specific strain of *Aspergillus*.

Concerning OTA production, cinnamon, thyme, citronella, and marjoram were effectuated lower than the limit of OTA quantification levels in both tested *A. carbonarius* strains when applied at the maximum concentration tested (100 μL/mL). Cinnamon, thyme, and citronella led to almost 100% OTA inhibition when applied at even lower concentrations (3 μL/mL, 6 μL/mL, and 20 μL/mL), indicating the strong potential of EOs in the prevention of OTA contamination in food products. As for *AclaeA* expression levels, cinnamon, thyme, and geranium, and to a lesser extent, citronella resulted in significantly reduced values, following the results of the inhibitory effect of the aforementioned EOs in OTA production. In a study by Lappa et al. (2017) [34], *laeA* did not show any differences in the transcriptional profile when EOs of clove, mandarin, and lemongrass were applied. Furthermore, eugenol (the major constituent in the aromatic oil extract from cloves) significantly reduced the transcription of the clustered genes for OTA biosynthesis in *A. carbonarius* in a study by Jiang et al. (2022) [18], confirmed by RT-qPCR. Some of the active compounds of EOs, such as thymol and eugenol, disrupt the cellular functions of fungi, leading to reduced viability and mycotoxin synthesis. Fennel, cardamom, anise, chamomile, celery, cinnamon, thyme, taramira, oregano, and rosemary inhibited the expression of the genes *acOTApks*, *acOTAnrps*, *acpks*, *laeA,* and *veA*; the *ackps* gene, for example, was downregulated by 99.2% when exposed to 5 µL/mL of fennel extract. Regarding the *laeA* gene, it was downregulated by 92%, 71%, and 80% by the corresponding extracts of fennel, chamomile, and rosemary [17]. Our results strongly support an underlying correlation between the effectiveness of the tested EOs in reducing OTA production and *AclaeA* transcription levels.

The mode of action of cinnamon oil against *A. carbonarius* and its ability to inhibit ochratoxin production can be attributed to several mechanisms: (1) cell membrane disruption by cinnamaldehyde, a key active component that can disrupt the integrity of fungal cell membranes; this disruption leads to increased permeability, causing the leakage of cellular contents and, ultimately, cell death; (2) inhibition of enzymatic activity by cinnamaldehyde and other components of cinnamon oil can that can hinder the activity of critical enzymes involved in the metabolic processes and OTA biosynthesis of *A. carbonarius*; (3) alteration of fungal gene expression, as has been proved by the downregulation of the *AclaeA* gene in the present study; (4) synergistic effects when combined with other essential oils or preservatives that enhance its antifungal activity against *A. carbonarius*. This could lead to more effective strategies for controlling mold growth and toxin production in agricultural products [17].

## 4. Conclusions

As confirmed by the findings of this study, the global regulator *AcLaeA* plays a key role in the physiology, virulence, and ochratoxin production of *A. carbonarius*. Until now, studies have shown that beneficial microorganisms, biostimulants, and essential oils can reduce OTA production by reducing fungal growth or/and conidia production by the fungus. The use of effective biopesticides Trianum-P^®^, Vacciplant^®^, and Serenade^®^ Max to decrease fungal growth and OTA contamination in vineyards showed strong potential to modulate the expression of *AcLaeA* in this study, indicating a novel possible control mechanism. Understanding *AcLaeA’s* role could lead to the optimization of biopesticide formulations to enhance their effectiveness in controlling *A. carbonarius* and OTA production. Another use of *AcLaeA* could be the development of novel antifungals, specifically aiming at OTA biosynthesis and its inhibition. By manipulating the expression of *AclaeA*, it might be possible to decrease the production of ochratoxins in agricultural settings, particularly in grapes and their products such as wine and raisins/currants. Additionally, the essential oils of cinnamon, geranium, and thyme showed promising results in downregulating the expression of *AcLaeA*. The application of EOs in agricultural practices can be explored as a natural alternative to synthetic fungicides. By incorporating these oils into post-harvest treatments or storage conditions, producers can potentially minimize the risk of OTA contamination in fruits and grains. Understanding the role of *AcLaeA* and its modulation opens new avenues for developing targeted strategies in the biological control of *A. carbonarius*. Further research is needed to elucidate the precise mechanisms underlying the interaction between *AcLaeA*, biocontrol agents, essential oils, and the physiology and virulence of *A. carbonarius*.

## 5. Materials and Methods

### 5.1. Strains and Growth Conditions

The ochratoxigenic *A. carbonarius* wild-type strain Ac ITEM 5010 was used in this study (NCBI: txid602072). The strain was cultured on Malt Extract Agar (MEA) media at 28 °C with 12 h photoperiod for 6 days to enable production of conidia. The fungal strain was stored and maintained at −80 °C as conidia suspension with 25% glycerol.

### 5.2. Gene Analysis

Using the orthologous *laeA* gene from *A. nidulans* as a starting point (Genbank ID CBF88745.1), the target *AclaeA* gene in *A. carbonarius* was identified. The sequence was utilized as query in an amino acid vs. translated nucleotide blast (tblastn) in JGI’s database for *A. carbonarius* (https://mycocosm.jgi.doe.gov/Aspca3/Aspca3.home.html) accessed on 30 January 2008. Many results were obtained from the blast analysis; the best sequencing result from the *A. carbonarius* database indicated a notably high similarity (66%) to the *laeA* gene from *A. nidulans* at nucleotide level.

### 5.3. Genomic DNA Extraction

Fungal cultures were grown for 2 days on Malt Extract Broth (MEB) (Lab-M, Heywood, UK) plates. Mycelium was lyophilized using a Freeze Dryer Lyophilizer FD-18-MD, Labfreez^®^, and 100 mg of the dried mycelium was mixed with 700 μL of LETS Buffer (20 mM of EDTA—pH 8, 0.5% SDS, 10 mM of Tris-Cl—pH 8, 0.1 M of LiCl) and left at room temperature (RT) for 5 min. A total of 700 μL of phenol: CHCL3: isoamyl alcohol (25:24:1) was added, and after 5 min inoculation at RT, the sample was centrifuged at 13,000× *g* for 10 min. The supernatant was transferred into a new 1.5 mL tube, and an equal volume of phenol: CHCL3: isoamyl alcohol (25:24:1) was added. After 10 min of centrifugation at 13,000 × *g,* the supernatant was transferred into a new 1.5 mL tube and mixed with 1 mL 95% EtOH. The DNA pellet was collected after 10 min of centrifugation at 13,000× *g* and then was washed with 500 μL of 70% EthOH. Finally, the pellet was air-dried, and the DNA was resuspended in 50 μL of 10 mM Tris Buffer pH 8 [36]. 

### 5.4. Plasmid Constructions

Standard methods were used for construction, maintenance, and isolation of recombinant plasmids. Fungal chromosomal DNA was isolated and analyzed from lyophilized mycelia, as described previously. The high copy number plasmid pBluescript II SK was used as backbone plasmid for the plasmids constructed. Specific primers were designed for the deletion of the gene from the genome of *A. carbonarius* (Table 2). Primers Acarb_DLaeA-F1-HindIII and Acarb_DLaeA-R1-EcoRI amplified a region of 934 kb upstream of the *AclaeA* gene, and primers DLaeA-F2-BamHI and DLaeA-F2-XbaI amplified a region of 1.040 bases downstream of the *AclaeA* gene. These regions were amplified by PCR with Taq polymerase (Qiagen, Hilden, Germany) using an annealing temperature of 55 °C. The geneticin gene cassette was used to replace *AclaeA* gene, as shown in Figure S2. The geneticin gene cassette in pBluescript II SK was cut with the restriction enzymes EcoRI and BamHI. *Escherichia coli* DH5α served as a host for the plasmid construct. The inactivation cassette was subcloned into the binary vector pGKO2, which was then inserted into the *Agrobacterium tumefaciens* strain AGL1. Plasmid extraction was carried out using the Plasmid miniprep kit (Qiagen, Hilden, Germany) according to the manufacturer’s protocol.

### 5.5. Fungal Transformation for Deletion of AcLaeA

Transformation of the *A. carbonarius* strain Ac-5010 [37] was carried out using 100 μL of conidia suspension of *A. carbonarius* (10^6^ conidia/mL) mixed with 100 μL of *A. tumefaciens* culture that were then plated on a sterile Hybond membrane for co-cultivation. After co-cultivation for two days, the Hybond membranes were transferred to a selection medium (Potato Dextrose Agar) containing the appropriate concentration of the antibiotic geneticin, cefotaxime (200 μM), and moxalactam (100 μg/mL) necessary to activate the suicide cassette HSVtk in the pGKO2 vector. After 3–4 days of incubation at 28 °C, the transformed colonies of the fungus were grown as a result of homologous genetic recombination [38].

### 5.6. Confirmation of Deletion of AcLaeA

Fungal DNA from the selected mutant strains was extracted as described previously and was used as template for further analysis. The deletion of the *AclaeA* gene was confirmed by PCR using the primers Acarb-LaeA-F4 and Acarb-LaeA-R2 primers (Table 1), amplifying a product of about 2000 bp within *AclaeA* gene. The PCR amplification conditions consisted of an initial denaturation step at 95 °C for 3 min; 39 cycles of (a) 95 °C for 30 s, (b) 55 °C for 30 s, and (c) 72 °C for 2 min; and a final elongation step at 72 °C for 10 min. RT-PCR was carried out by using the Acarb-LaeA-F1-RT and Acarb-LaeA-R2 primers (amplify a product of about 750 bases within *AclaeA* gene) in order to verify the deletion of the *AclaeA* gene (Table 2).

### 5.7. AclaeA Gene Expression Analysis

MEB liquid cultures of wild-type and Δ*AcleaA* strains in Petri dishes were inoculated with 100 μL of a conidial suspension (10^6^ conidia/mL) and sub-cultured at 28 °C under darkness. The mycelium was collected at 48 h, 72 h, and 96 h after inoculation, frozen in liquid nitrogen, and stored at −80 °C before nucleic acid extraction. Total RNA was extracted from 100 mg of ground mycelium using the TRIzol™ Reagent extraction protocol (Thermo Fischer Scientific Inc., Waltham, MA, USA). The extracted RNA was resuspended using 50 μL of RNase free water. The RNA concentration (ng/μL) was measured by Nanodrop (Thermo Fisher Scientific Inc., Waltham, MA, USA). Total RNA was treated with DNase I, RNase-free (Fermentas—Thermo Fisher Scientific Inc., Waltham, MA, USA) to remove contaminating genomic DNA. Single-strand cDNA was synthesized from 5 μg of total RNA using Superscript II Reverse Transcriptase reverse transcription kit and an oligo (dT) according to the manufacturer’s instructions (Invitrogen—Thermo Fisher Scientific Inc., Waltham, MA, USA). Then, in order to further study the expression of *Ac*-*LaeA* gene over time, the specific primers Acarb-LaeA-F3-RT and Acarb-LaeA-R3-RT were designed with Primer 3 Software [39]. Real-time PCR reaction was performed on the Mx3005PTM (Stratagene, La Jolla, CA, USA) thermocycler to monitor cDNA amplification. The primer pair tubF and tubR was designed within the beta-tubulin gene to use it as a reference gene. The thermocycler protocol included one cycle at 98 °C for 2 min, followed by 40 cycles at 98 °C for 5 s and 55 °C for 30 sec. The expression of *AclaeA* gene was calculated using the absolute quantification method via normalization with absolute quantification of beta-tubulin gene expression using the 2^−∆Ct^ formula or 2^(Ct internal standard)−(Ct target)^ [40,41]. Gene expression analyses were derived from three biological triplicates.

### 5.8. Physiological Studies of ΔAcleaA Strains

Three Δ*AclaeA* strains were selected for growth assessment in comparison with the wild-type strain Ac-5010. PDA and MEA plates were inoculated with 10 μL of conidia suspension (10^6^ conidia/mL) of the wild-type strain Ac-5010 or the Δ*AclaeA* strains in the center of plates containing 20 mL of each medium. Cultures were incubated at 28 °C under light and dark conditions. The diameter of the growing colonies was measured daily for 7 days. Production of wild-type and Δ*AclaeA* strains conidia was also counted in the 7-day old fungal colonies. Three agar plugs (0.5 cm in diameter and 0.3 cm high) were removed from the outer part of the colonies and homogenized, and the conidia concentration was counted using a hemocytometer.

### 5.9. In Vitro Ochratoxin A Quantification

In order to quantify the ochratoxin production of the Ac-5010 wild-type strain and the three Δ*AclaeA* mutants (TMI1.4, TMI1.8, and TMI1.9), all strains were cultured on MEA and MEB nutrient media for 7 days at 25 °C under darkness. Specifically, at the end of storage, the entire content of each Petri dish or tube (biomass + substrate) was weighed and homogenized with an extraction solution of 80% methanol: 20% water (ratio 1:4 sample to solvent) in an Ultra Turrax homogenizer (Heidolph Instruments, Schwabach, Germany) for 2 min at maximum speed (26 × 10^3^ rpm). Extracts were filtered through Whatman No. 1 and subsequently through Glass acrodisc (0.22 μm) filters (n = 2). The detection and quantification of OTA was performed via HPLC according to the protocol outlined by Kapetanakou et al. (2011) [11]. Samples containing low OTA concentrations (Δ*AclaeA* strains) were purified and condensed using immunoaffinity columns (Ochrastar™, Romer Labs Pte Ltd., Singapore), while samples that had high concentrations (wild strains) outside the calibration curve were diluted with the mobile phase prior to HPLC analysis. A calibration curve was determined in the range of 0–500 ppb, starting from a dense solution of OTA (10.15 μg/mL, Biopure, Romer Labs^®^, Getzersdorf, Austria), while recovery percentages of OTA (2, 10, 50, 125, 250, and 500 ppb) were determined in the range of 97–136%.

### 5.10. Pathogenicity Assays on Grapes

The role of the *AclaeA* gene in the pathogenicity and virulence of *A. carbonarius* was further investigated by testing the ability of *Ac*-5010 wild strain and Δ*AclaeA* strains TMI1.4, TMI1.8, and TMI1.9 to cause disease in three different grape varieties: *cv*. Fraoula, *cv*. Soultanina, and *cv*. Moschato. Three replicates were used for each pathogenicity test, and each replicate consisted of 8 berries. Artificial inoculation was performed using 40 μL conidia suspension (10^4^ conidia/mL) on each slightly wounded fruit. Control grape berries were treated with sterilized water. After inoculation, the berries were placed in plastic sterile transparent boxes and kept at 28 °C with a 12 h photoperiod. After 6 days, infected grape berries were placed in 50 mL Falcon tubes and homogenized in sterile water, and the amount of conidia was measured per berry.

### 5.11. Biocontrol Agents and Expression of AcLaeA

The commercial biological Plant Protection Products (bioPPPs) Vacciplant^®^ (20 mL/10 lt), with active ingredient laminarin; Trianum-P^®^ (3 gr/10 lt), with active fungal ingredient *T. harzianum* T-22; and Serenade^®^ Max (10 mL/lt), with active bacterial ingredient *B. subtilis* (strain QST 713), showed a decrease in *A. carbonarius* grape black and sour rot disease and OTA biosynthesis in vineyards (unpublished data from D.I. Tsitsigiannis research group). MEB Petri dishes with 20 mL of media each and amended with each bioPPP were inoculated with a conidial suspension (10^6^ conidia/mL) of the wild-type *A. carbonarius* strain Ac-5010 and incubated at 25 °C under darkness. As control treatments, plates with only the Ac-5010 strain were used. The mycelium was collected after 48 h, 72 h, and 96 h; frozen immediately in liquid nitrogen; and stored at −80 °C before nucleic acid extraction. RNA was extracted according to the TRI Reagent^®^ RT Protocol by Molecular Research Center (MRC Inc., Cincinnati, OH, USA). Total RNA was treated with DNase I (RNase-free, NEB Biolabs, Cambridge, UK) to remove the genomic DNA. Single-strand cDNA was synthesized from 5 μg of total RNA using Firescript RT cDNA Synthesis Kit according to the manufacturer’s instructions (Solis Biodyne, Tartu, Estonia). The specific primers Acarb-LaeA-F3-RT and Acarb-LaeA-R3-RT (Table 1) were used for gene expression analysis of *AclaeA*. Real-time PCR reaction was performed with KAPA SYBR^®^ Fast Universal qPCR Kit (KAPA Biosystems, UK). The primer pair tubF and tubR were used for gene expression analysis of the beta-tubulin gene as a reference gene. The expression of *AclaeA* gene after the different treatments was calculated using the absolute quantification method via normalization with the absolute quantification of the beta-tubulin gene expression using the 2^−∆Ct^ formula or 2^(Ct internal standard)−(Ct target)^ [40,41]. Gene expression analyses were derived from three biological triplicates.

### 5.12. In Vitro Evaluation of Ten Essential Oils in Inhibiting Mycelial Growth and Conidia Production in Aspergillus Carbonarius

Ten essential oils (EOs) from the following plants were used in these studies: *Cinnamomum zeylanicum*, *Cymbopogon nardus*, *Lavandula angustifolia*, *Melaleuca alternifolia*, *Mentha piperitha*, *Origanum majorana*, *Pelargonium gaveolens*, *Rosmarinus officinalis*, *Salvia sclarea,* and *Thymus vulgaris*. The EOs were obtained from APIVITA (Greece). For each EO, appropriate volumes were added to two different media, Malt-Extract-Agar (MEA) and Czapek-Yeast-Agar (CYA) [17] at approximately 40–50 °C in amounts needed to achieve the concentrations of 2 μL EOs/mL of medium, 3 μL/mL, 6 μL/mL, 20 μL/mL, and 100 μL/mL. A 10 μL drop of conidial suspension with a concentration of 10^6^ conidia/mL was obtained from *A. carbonarius* wild-type strains Ac-5010 and Ac-29 [42] colonies (7 days MEA cultures) and then placed in the centers of two 9 cm diameter Petri dishes containing MEA and CYA, respectively, amended with each tested EO and concentration. Cultures were incubated at 28 °C for 7 days, and colony diameter (cm) was measured daily from the 2nd day of the inoculation until the last day of the experiment and expressed as a percentage of the diameter of the controls, which were unmodified MEA and CYA plates, allowing the optimum growth of the fungal isolates. On the last day of the experiment (7th day post-inoculation), the number of the produced conidia by each *A. carbonarius* strain was determined, and the result was expressed as the percentage of produced conidia of the positive control for each media. Produced conidia were observed via microscopy. There were three replicated dishes for each combination of EO, concentration, and strain, and the experiment was repeated twice.

### 5.13. In Vitro Evaluation of the Essential Oils in OTA Production

The quantification of OTA produced by each mycotoxigenic *A. carbonarius* strain on MEA described before *(2.6)* for each EO was conducted using the Romer Labs (Getzersdorf, Austria), AgraQuant^®^ ELISA Ochratoxin Assay kit (2–40 ppb) 7 days post-inoculation on MEA plates. OTA extraction was conducted according to the manufacturer’s instructions.

### 5.14. In Vitro Evaluation of Essential Oils in AclaeA Expression Levels

Based on the effectiveness in reducing *A. carbonarius* growth and OTA production via Ac-5010, the EOs *C. zeylanicum* (2 μL mL^−1^), *C. nardus* (6 μL mL^−1^), *M. piperitha* (3 μL mL^−^^1^), *P. gaveolens* (3 μL mL^−^^1^), *R. officinalis* (3 μL mL^−^^1^), and *T. vulgaris* (3 μL mL^−^^1^) and their optimum concentrations, respectively, were chosen for the in vitro evaluation of the expression of *AclaeA* expression levels. Malt Extract Broth (MEB) liquid media amended with each EO and the corresponding dose were inoculated with a conidial suspension with a 10^6^ conidia/mL concentration of WT strain Ac-5010. Liquid cultures were incubated at 25 °C in dark conditions, and sampling took place on the 2nd, 3rd, and 4th day post-inoculation. The collected sample was immersed in liquid nitrogen and stored at 80 °C until used for further analysis. Prior to RNA extraction, the samples were lyophilized for 12 h using a Freeze Dryer Lyophilizer FD-18-MD, Labfreez^®^. RNA extraction was conducted using TRI Reagent^®^ RT Protocol by Molecular Research Center (MRC Inc., Cincinnati, OH, USA). The extracted total RNA was used as a template for RT-qPCR, and the cDNA was created using PrimeScript™ RT Reagent Kit (Perfect Real Time), Takara Bio Inc. (Kusatsu, Shiga, Japan), according to the manufacturer’s instructions. The RT-qPCR reactions were performed using a StepOne Plus Real-Time PCR System (Applied Biosystems, Waltham, MA, USA) using an SYBR Green based kit (KAPA SYBR^®^ FAST qPCR, Master Mix (2X) Kit, Kapa Biosystems, Wilmington, DE, USA) according to the manufacturer’s instructions. The amplification conditions were 55 °C for 10 min; and 95 °C for 2 min, followed by 40 cycles of 95 °C for 10 s and 60 °C for 1 min, while the melt curve stage consisted of 95 °C for 15 s, 60 °C for 1 min and 95 °C for 15 s. The threshold cycle (Ct) was determined using the default threshold settings. The 2^−∆∆Ct^ method was applied to calculate the relative gene expression levels. The tubulin gene (tub) was used as the endogenous control. The primers that were used for the RT-qPCR were tub-F and tub-R for the housekeeping gene and A_carb_LaeA_F3-RT and A_carb_LaeA_R3_RT (Table 1) for *AclaeA* gene. There were three replicated samples for each EO, and the experiment was conducted two times.

### 5.15. Statistical Analyses

All the data were normalized via square root transformation and then subjected to ANOVA followed by Tukey’s Honest Significant Difference (HSD) test as a post hoc test (Statgraphics Centurion XVI, Version 16.1.11). Differences at *p* ≤ 0.05 were considered significant.

## Figures and Tables

**Table 1:** EC_50_ values for each strain, media, and essential oil (μL mL*^−^*^1^).

Common Plant Name	Plant Species	MEA		CYA	
		Ac5010	Ac-29	Ac5010	Ac-29
Cinnamon	*Cinnamomum zeylanicum*	1.48	2.77	<2	1.19
Citronella	*Cymbopogon nardus*	13.07	10.98	9.02	9.66
Lavender	*Lavandula angustifolia*	48.82	69.11	61.16	67.60
Tea tree	*Melaleuca alternifolia*	27.16	65.28	55.84	50.04
Mint	*Mentha piperitha*	16.90	13.86	49.94	6.74
Marjoram	*Origanum majorana*	22.00	73.53	>100	50.14
Geranium	*Pelargonium gaveolens*	8.32	11.75	13.51	27.74
Rosemary	*Rosmarinus officinalis*	27.47	>100	>100	96.97
Clary Sage	*Salvia sclarea*	61.67	89.62	68.15	59.92
Thyme	*Thymus vulgaris*	3.14	3.56	3.35	2.52

**Table 2:** List of primers used in this study. A. Primers used for the construction of the cassette for the deletion of the *AclaeA* gene in *A. carbonarius*. B. Primers used for the verification of the deletion of the *AclaeA* gene in *A. carbonarius*. C. Primers used for the expression of the *AclaeA* gene in *A. carbonarius* via real-time PCR.

	Primer Name	Sequence (5′→3′)
A	Acarb_DLaeA-F1-HindIII	5′-ATCTTCCCAAGCTTCCAAGTTCCC-3′
	Acarb_DLaeA-R1-EcoRI	5′-GCGCGCGAATTCGGAGGCTATGTGTCAGAGG-3′
	DLaeA-F2-BamHI	5′-GCGCGCGGATCCTCAACGCGAGACCGATACAAT-3′
	DLaeA-F2-XbaI	5′-GCGCGCCTCGAGCGGACAGCGAAAGCGAGGATGA-3′
B	Acarb-LaeA-F4	5′-CTGCTTCCCCAACTCTCTTTC-3′
	Acarb-LaeA-R2	5′-GCGACCTTTCTCTCATGCTC-3′
	Acarb-LaeA-F1-RT	5′-TGGGCGACTAATCTTGGAAC-3′
C	Acarb-LaeA-F3-RT	5′-TATGCGCCATTTGATTTTGA-3′
	Acarb-LaeA-R3-RT	5′-GAAGGTCTCTGACGGTGTCC-3′
	*tubF*	5′-TTCCCCCGTCTCCACTTCTTCATG-3′
	*tubR*	5′-GACGAGATCGTTCATGTTGAACTC-3′

## Data Availability

The datasets generated during and/or analyzed during the current study are available from the corresponding author upon reasonable request.

## Supplementary Materials

The following supporting information can be downloaded at: https://www.mdpi.com/article/10.3390/toxins17010002/s1, Figure S1: Confirmation of Δ*AcLaeA* mutant strains; Figure S2: Design of allelic replacement of the *AclaeA* gene by geneticin.
