# Supplementary material for: The Global Secondary Metabolite Regulator AcLaeA Modulates Aspergillus carbonarius Virulence, Ochratoxin Biosynthesis, and the Mode of Action of Biopesticides and Essential Oils
Source: Toxins (Basel). 2024 Dec 24;17(1):2. doi: 10.3390/toxins17010002 (PMC11768970; doi:10.3390/toxins17010002)
Supplement: Supplementary file 1 [file toxins-17-00002-s001.zip › toxins-3322002-supplementary.pdf]

Supplementary Material

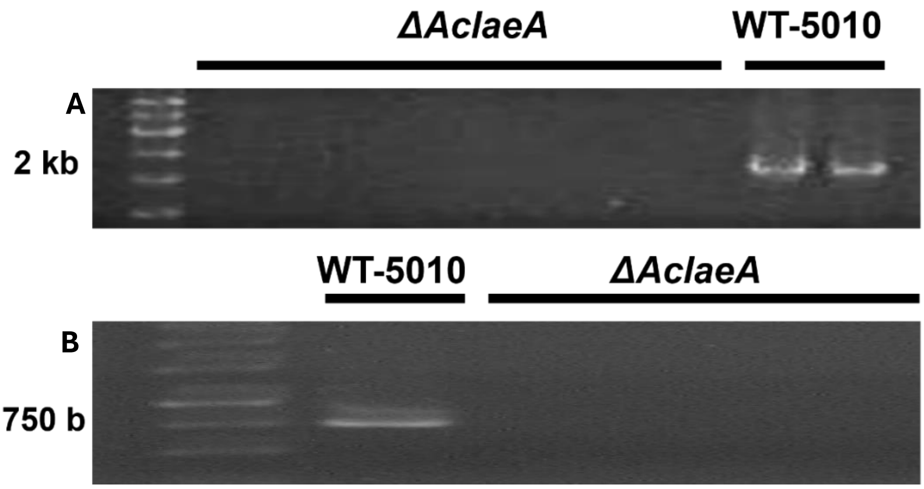

Figure S1. Confirmation of Δ*AcLaeA* mutant strains. **A.** PCR with the primers Acarb-LaeA-F4 and Acarb-LaeA-R2 showed the expected product (1997 bases) only in the WT Ac-5010. **B.** PCR with the primers Acarb-LaeA-F1-RT and Acarb-LaeA-R2 primers showed the expected product (750 bases) only in the WT strain Ac ITEM 5010.

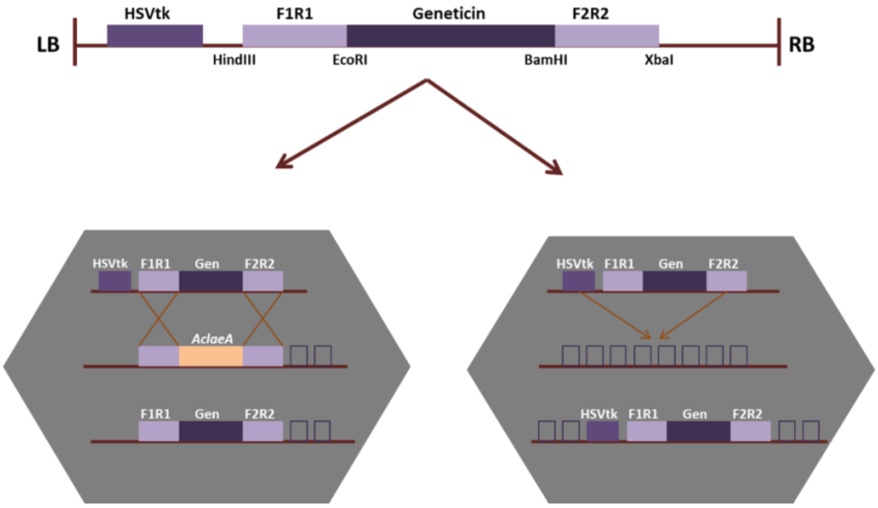

Figure S2. Design of allelic replacement of the *AclaeA* gene by geneticin. RB, LB: RB-right border and LB-left border of the Ti plasmid (tumor-inducing plasmid) of *Agrobacterium tumefaciens*.
